# A Comprehensive Review of Chemistry, Sources and Bioavailability of Omega-3 Fatty Acids

**DOI:** 10.3390/nu10111662

**Published:** 2018-11-04

**Authors:** Mateusz Cholewski, Monika Tomczykowa, Michał Tomczyk

**Affiliations:** 1 Department of Pharmacognosy, Faculty of Pharmacy, Medical University of Białystok, ul. Mickiewicza 2a, 15-230 Białystok, Poland; matchol.bot@gmail.com; 2 Department of Organic Chemistry, Faculty of Pharmacy, Medical University of Białystok, ul. Mickiewicza 2a, 15-230 Białystok, Poland; monika.tomczyk@umb.edu.pl

**Keywords:** Omega-3 fatty acids, chemistry, sources, bioavailability

## Abstract

Omega-3 fatty acids, one of the key building blocks of cell membranes, have been of particular interest to scientists for many years. However, only a small group of the most important omega-3 polyunsaturated fatty acids are considered. This full-length review presents a broad and relatively complete cross-section of knowledge about omega-3 monounsaturated fatty acids, polyunsaturates, and an outline of their modifications. This is important because all these subgroups undoubtedly play an important role in the function of organisms. Some monounsaturated omega-3s are pheromone precursors in insects. Polyunsaturates with a very long chain are commonly found in the central nervous system and mammalian testes, in sponge organisms, and are also immunomodulating agents. Numerous modifications of omega-3 acids are plant hormones. Their chemical structure, chemical binding (in triacylglycerols, phospholipids, and ethyl esters) and bioavailability have been widely discussed indicating a correlation between the last two. Particular attention is paid to the effective methods of supplementation, and a detailed list of sources of omega-3 acids is presented, with meticulous reference to the generally available food. Both the oral and parenteral routes of administration are taken into account, and the omega-3 transport through the blood-brain barrier is mentioned. Having different eating habits in mind, the interactions between food fatty acids intake are discussed. Omega-3 acids are very susceptible to oxidation, and storage conditions often lead to a dramatic increase in this exposure. Therefore, the effect of oxidation on their bioavailability is briefly outlined.

## 1. Chemistry of Omega-3 Fatty Acids

Omega-3 fatty acids, called *n*-3 fatty acids or ω−3 fatty acids (*n*-3 FAs), are a heterogeneous group of fatty acids with a double bond between the third and fourth carbon atoms from the methyl end (from the ω−1 carbon atom). In general, we distinguish among them monounsaturated fatty acids (MUFAs; one double bond in carbon chain) and polyunsaturated fatty acids (PUFAs; more than one double bond in carbon chain). Conjugated fatty acids (CFAs) are a subset of PUFAs with at least one pair of conjugated double bonds [1], i.e., the double bonds are not separated by methylene bridges, but one single bond. We also mention some examples of modified omega-3 fatty acids like hydroxy fatty acids (HFAs), oxo fatty acids (keto fatty acids) and hydroperoxy fatty acid. Among the hydroxy fatty acids, we distinguish saturated or unsaturated fatty acids, consisting of a long unbranched carbon chain with a carboxyl group at one end and one or more hydroxy groups. Oxo or keto fatty acids are fatty acids having both a carboxy group and a ketonic or aldehydic group in the molecule. Hydroperoxy fatty acids, in turn, carry at least one hydroperoxy group (-OOH) in the molecule. Some authors find the terms long-chain (LC) *n*-3 PUFAs and omega-3 fatty acids identical in meaning [2], which can be misleading because “omega-3 fatty acids” is a broader term.

We assumed all fatty acids with a double bond at the ω−3 carbon atom to be omega-3 fatty acids. Omega-3 fatty acids show *cis*-*trans* isomerism with its extension to *E*-*Z* configuration [3]. We can speak of geometrical isomerism in the case of omega-3 fatty acids because two carbon atoms with sp2 hybridization connected by a double bond are linked to a hydrogen atom and group of atoms each. In order to determine the type of geometrical isomerism, at the beginning we choose the two most important substituents—one on the left, the second on the right of the double bond. In fatty acids, we have only one group (of atoms) on each side, because the two remaining binding sites occupies a hydrogen atom. In the *cis*-isomer these two groups are located on the same side of the reference plane (the plane passing through the atoms connected by a double bond and perpendicular to the plane in which these atoms and atoms directly associated with them are situated); in the *trans*-isomer they are in contrary positions [4]. The *E*-*Z* system is a bit more detailed. The mutual placement of the substituents is described by the Cahn-Ingold-Prelog (CIP) rule. The most important is the substituent whose atom directly connecting to the rest of the molecule (directly with the atom forming the double bond) has a higher atomic number (in the case of isotopes, a higher atomic mass). If in this position, in the substituents (on the right or left side of the double bond), there are identical atoms, then (to choose a substituent of greater importance) we take into account subsequent atoms, always choosing atoms with the highest atomic number. If a given atom is connected by multiple bonds, the bond should be replaced by the number of single bonds appropriate for its multiplicity—each atom present at a multiple binding must after transformation have a corresponding number of single bonds (C=C = 2 × C-C). The “*E*” configuration (from entgegen, German for “opposite”) means that two groups of higher CIP priority (one on the left, the second on the right from the double bond) are on opposite sides of the double bond (in the synperiplanar position). If those groups are on the same side of the double bond (in antyperiplanar position), configuration is defined as “*Z*” (from zusammen, German for “together”) [5]. For simplicity, according to many authors, we used the terms “*cis*” and “*Z”* as well as “*trans*” and “*E”* interchangeably. The *cis-trans* isomerism of fatty acids seems to play a particularly important role in shaping their chemical and biological activity, a good example of which are the various properties of conjugated fatty acid isomers [1].

Naturally occurring fatty acids usually have from four to 28 carbon atoms. However, many of them, especially those found in the brain, retina and spermatozoa, have a longer carbon chain [6,7,8]. Fatty acids can be divided, depending on the length of the carbon chain, into four basic groups:

- Short-chain fatty acids (SCFAs), sometimes called volatile fatty acids (VFAs), contain from one to six carbon atoms (C1–6), formed as a result of the fermentation of carbohydrates by the gut microbiota in the digestive tract of mammals [9].
- Medium-chain fatty acids (MCFAs) have from seven to 12 carbon atoms (C7–12) [10]; according to other sources eight to 14 carbon atoms [11].
- Long-chain fatty acids (LCFAs) have from 14 to 18 carbon atoms (C14–18) and constitute the majority of fatty acids taken with food (diet) [12].
- Very long-chain fatty acids (VLCFAs) have backbones containing more than 20 carbon atoms (C > 20) [13], or according to other authors no fewer than 20 carbon atoms (C ≥ 20) [12] or even more than 22 carbon atoms (C > 22) [14,15,16].

There can also be distinguished fatty acid subgroups, such as dietary long-chain saturated fatty acids (C ≥ 16) and long-chain polyunsaturated fatty acids (LCPUFAs/LC PUFAs; C ≥ 18). While dietary long-chain saturated fatty acids do not directly concern the subject of this article, they deserve to be distinguished in the general chemical classification due to the ease of incorporation into the adipose tissue, and therefore, *nomen omen*, special dietary significance [11]. Fatty acids with nine or fewer carbon atoms are in a liquid state at room temperature [10].

The most important, but small, group of fatty acids for humans are essential fatty acids (EFAs), which are necessary to maintain homeostasis, cannot be synthesized, or rather cannot be synthesized sufficiently by the organism, and must be supplied with food [17,18]. The significance of fatty acids in the animal diet was discussed by Osborne and Mendel [19] in 1920. In 1929 Burr and Burr [20] proved in their experiments on rats the ‘essential’ nature of some fatty acids. Some authors consider all PUFAs to be essential fatty acids [21] and determine linoleic acid (LA) and *alpha*-linolenic acid (ALA) as the most important [17,22], calling them “parent essential fatty acids” [23,24]. Others considered only arachidonic and linoleic acids as essential fatty acids because of their importance for the body’s growth and for maintaining the integrity of the skin [21]. The mammalian literature indicates 23 acids as essential, while the aquatic literature quotes only two EFAs—EPA and DHA. Considering the importance of ARA, we can take ARA, DHA and EPA as the most important long-chain PUFAs in mammals and fish [25]. Some animals can synthesize them using LA and ALA as precursors [25,26]. However, those precursors must be available in sufficient quantities [25]. Gurr and Harwood [27] detailed “essential nutrients” in contrast to “essential metabolites”. In the light of this assumption, “essential nutrients” are precursors of “essential metabolites” and cannot be synthesized by organisms for which they are “essential”. Notwithstanding, “essential” is a relative term—human and rats can synthesize LA and ALA from 16:2*n*-6 and 16:3*n*-3 contained in green vegetables, i.e., in microalgae, in conditions of defined substrate availability [28,29,30,31]. Considering that there is insufficient data showing that any individual PUFA is absolutely necessary during life, Cunnane divided essential fatty acids into ‘conditionally indispensable’ and ‘conditionally dispensable’ [32,33,34].

The most important data concerning chemistry and sources of omega-3 fatty acids are included in Table 1.

## 2. Sources of Omega-3 Fatty Acids 

In the years 2003–2008 people in the US consumed, with food, on average 0.17 g/day (median intake 0.11 g/day) of long-chain omega-3 fatty acids (DHA, EPA and EPA equivalents (5% from conversion of ALA, 33% from conversion of SDA)), i.e., lower than the recommended 0.5 g/day. Among people consuming omega-3 fatty acids in the group below or at the 15th percentile of omega−3 FAs total consumption, the most important source of omega-3 acids were cereal products (36%), while in the group above or at the 85th percentile, the dominant source was fish (fish and mixtures; 71%—in contrast to the low-intake group, where the fish consumption was 1%). In both groups, the intake of seeds and nuts was low, and vegetables were consumed at 9% and 2%, respectively [134]. As can be easily seen, the share of omega-3 fatty acids in the diet is most affected by the supply of fish, because they are the primary source of EPA and DHA for humans [135]. This is due to the fact that the food of many fish are algae rich in EPA and DHA, and other organisms consuming algae, like fish or marine invertebrates [136,137]. Microalgae play a key role in the primary production of PUFAs and are their main source in seawater. Marine invertebrates are also an important and basic source of omega-3 PUFAs due to their ability to synthesize some of them de novo, for example, oyster *Crassostrea gigas* can produce EPA and DHA by consuming microalgae that do not contain both [137]. Seafood contains many valuable omega-3 acids. However, their frequent consumption exposes the human body to the neurotoxic effect of methyl mercury, which is especially harmful for the development of the central nervous system of the fetus [138]. Therefore, it is advisable to look for other sources of these fatty acids, and to include them in a balanced diet.

EPA occurs in large amounts in herring (15% of total lipids) [59], wild sardine (13.6% of muscle total FAs) [61] and pollock roe (18.8%) [59]. In plants, it is rather rare. Mention should be made of *Undaria pinnatifida* (13% of essential oil composition) [107] and *Rhododendron sochadzeae* (2% of leaf FA hexan extract) [108].

The main sources of DHA are flyingfish, herring, pollock and salmon roe (27.9%, 22.6%, 22.2%, and 17.4% of total lipids, respectively) [59], *Cirrhinus mrigata* (18.07 g/100 g muscle tissue FAs), and *Catla catla* (17.98 g/100 g muscle tissue FAs) [58]. The presence of this acid in the jackalberry (4.65 g FA per 100 g oil *Diospyros mespiliformis*) is noteworthy [139]. 

As mentioned above, both EPA and DHA are also synthesized from ALA using enzymes—desaturases and elongases [17,22,26,134], although in humans this process is insufficient—the conversion rate is 10–14% for men and women, respectively [136,140]. According to other authors, ALA is converted to EPA with a yield of 0.2 to 21% (in adult men 8%), and to DHA of zero to 9% (in women more than 9%) [141]. DHA can also be a substrate for the creation of EPA and vice versa (the conversion efficiency of EPA to DHA is < 0.1% in adult men) [141,142]. Moreover, ALA is a precursor of DPA as well [143]. 

*α*-Linolenic acid (ALA) is common in the higher plants and algae [22]. Rich sources of ALA also include *Linum usitatissimum* (depending on the genotype it contains from 1.1 to 65.2% of total fatty acids in seeds) [71], chia *Salvia hispanica* (64.04% of seed oil fatty acids and 16.4 g/100 g of milled chia seeds) [65,68], *Trichosanthes kirilowii* (33.77–38.66% of seed oils) [70], paprika *Capsicum annuum* (29.93% of fresh pericarp fatty acids in the Jaranda variety and 30.27% in the Jariza variety) [72], and many others. The content of ALA in fish is small, for example 1.1% in wild sardine (*Sardina pilchardus*) muscle total FAs [61].

8(*Z*),11(*Z*),14(*Z*)-Heptadecatrienoic acid, also called norlinolenic acid, was identified in *Salvia nilotica* [0.4% of mixed esters and 2.3% of IV fraction (% by gas liquid chromatography)] [55].

(all *trans*)-9,12,15-Octadecatrienoic acid (linolenelaidic acid) is present in Turkish sage species, and the weight per cent of seed FAs of *Salvia virgata*, *Salvia potentillifolia*, *Salvia recognita*, *Salvia tomentosa* amounts to 0.4, 0.7, 1.1, and 1.4%, respectively [73]. *Nicotiana tabacum* also contains linolenelaidic acid but in a minor amount, which is 0.03% of tobacco seed oil FAs [74].

(9*Z*,12*Z*,15*Z*)-2-Hydroxyoctadeca-9,12,15-trienoic acid (2-hydroxylinolenic acid, *alpha*-hydroxylinolenic acid) is a little known hydroxy acid found in *Salvia nilotica* (5.4% of *Salvia nilotica* seed oil) [55].

(11*Z*,14*Z*,17*Z*)-Icosa-11,14,17-trienoic acid (eicosatrienoic acid), sometimes named homo-*alpha*-linolenic acid, rarely occurs in animal sources—hen egg yolk contains this acid in the amount of only 0.15 – 0.16% of total lipids [101]. Much more was found in *Torreya grandis* kernel oil (6.78–8.73% of total FAs) [96] and cork *Phellodendron lavalei* seeds [15.5% of free fatty acids (FFAs)] [98], but the largest amount is contained in *Pittosporum undulatum* seed oil (31.44% of total FAs) [97].

Close derivatives of alpha-linolenic acid are two acids included in the minor polyenoic acids group [75]. Rumelenic acid (*cis*-9, *trans*-11, *cis*-15-octadecatrienoic acid) is formed by the isomerization of *alpha*-linolenic acid, while *cis*-9, *trans*-11, *trans*-15-octadecatrienoic acid is the isomer of rumelenic acid. Both acids are found in ruminants, for example in sheep’s milk [69,75].

Docosapentaenoic acid (DPA; (7*Z*,10*Z*,13*Z*,16*Z*,19*Z*)-docosa-7,10,13,16,19-pentaenoic acid), tradinionally called clupanodonic acid, shows structural similarity to EPA but has two more carbon atoms in the chain. Significant amounts are present in meat (13.8/16.7% of wether/ewe (lamb) raw meat FAs) [116], fish roe (5.5% of salmon roe total lipids [59], fish oils (1.04–2.82% of European wels catfish oil) [52], seal oil (2.64–4.74%) [62], and fish muscle (1.6% of wild sardine (*Sardina pilchardus*) muscle total fatty acids) [61]. DPA sporadically occurs in algae such as *Hormosira banksii* and *Dictyota dichomota* (0.07% and 1.6% of total FAs, respectively) [117].

Stearidonic acid (SDA; moroctic acid) is common in plants and fishes. Salmon roe contains a small SDA portion (0.8% of total lipids) [59]; a little more in wild sardine (2.3% of muscle total FAs) [61]. SDA occurs in many plants but in small amounts, except for plants from the families Primulaceae and Boraginaceae, where it can be found in relative big concentrations (mean amounts of 4.73 and 4.99% of seeds total FA methyl esters, respectively). *Hippophae rhamnoides* (Elaeagnaceae) seeds contain only 0.36% SDA [78]. Particularly noteworthy is *Echium humile* ssp. *pycnanthum*, in which seeds had SDA content estimated at up to 16.2% of total FAs [79]. 

(7*Z*,10*Z*,13*Z*)-Hexadeca-7,10,13-trienoic acid (roughanic acid) occurs in many plants and in fish oils, but usually in small amounts. As a result of reactions catalyzed by lipoxygenases, this is converted to oxygenated metabolites such as 7-, 8-, 10-, 11-, 13-, 14-hydroperoxides, and bis-allylic 9-hydroperoxide. However, hydroxy derivatives of roughanic acid are also present in plants rich in alpha-linolenic acid and not roughanic acid, including soybean and pea seedlings [144].

Omega-3 monounsaturated fatty acids are a small group, although with an undeniable role in nature—some such as (*Z*)-11-tetradecenoic acid, (*E*)-11-tetradecenoic acid and (10*E*,13*Z*)-10,13-heksadecadienoic acid are pheromone precursors in insects [38]. As contained in generally available food sources, 13*Z*-hexadecenoic acid, 15*Z*-octadecenoic acid, 9*E*-dodecenoic acid and 11*E*-tetradecenoic acid are especially worth noting. 15*Z*-octadecenoic acid, found in beef [46], pork [45], cows milk and butter [47], and mature human milk, is relatively common [49,50]. 11*E*-tetradecenoic acid occurs in *Coriandrum sativum*, whose leaf essential oil contains 13.37% of this acid [39]. 

Conjugated fatty acids still require detailed research, as some of them (conjugated linoleic acid) have antitumor activity and a number of properties that give benefits in the fight against hypertension, obesity and diabetes [1,145]. Conjugated omega-3 fatty acids, important from the point of view of their availability in food, are *alpha*- and *beta*-parinaric acid. *Trans*-parinaric acid, also called *beta*-parinaric acid [88], has all the bonds in the *trans* configuration. In the case of *cis*-parinaric acid (*alpha*-parinaric acid [146]), however, the name is somewhat misleading, because its isomerism can be described as *cis-trans-trans-cis* [147]. Parinaric acid, due to its natural ability of fluorescence and high susceptibility to free radicals, is used (as *cis*-parinaric acid) as an indicator of lipid peroxidation in cell membranes [148,149,150,151,152,153,154] and (as *trans*-parinaric acid) to assess the effect of temperature change on cell membranes [88,155]. Some organisms are able to convert nonconjugated fatty acids into conjugated counterparts [156]. In addition to tetraenoic parinaric acid, in plants mainly conjugated trienoic acids are found with 18 and 17 carbon atoms. Acetylenic and dienoic ones may have a different number of methylene bridges [157]. *Parinarium laurinum* seeds are the richest in alpha-parinaric acid sources (62% of seed oil methyl esters) [81]. 

Very long-chain fatty acids (VLCFAs; C > 22) play many important roles in the body, including modulating the functions of neutrophils. While their activity decreases with increasing carbon chain length, they remain efficient superoxide production activators in neutrophils, much better than *N*-formylmethionyl-leucyl-phenylalanine (FMLP) [15]. VLCFAs also have the ability to regulate PKC activity [16]. However, this still requires further research, especially in vivo. Their predisposed location in the body is also interesting—they are found in large amounts in the human brain [7,122,158,159], bovine retina [8,122], ram, bull, boar, and human spermatozoa [7,14] as well as in rat testicles [160], cultures of mouse spermatides and spermatocytes [161], and human vascular endothelial cells culture [162]. The VLC *n*-3 FAs acids have been found in dipolyunsaturated phosphatydylcholines of bovine retina and sphingomyelin of ram spermatozoa, as well as in bull spermatozoa [7,8,14]. In the normal human brain, the presence of VLC *n*-3 hexaenoic acids (36:6) was also found, but in smaller amounts than omega-6 fatty acids [122]. Tetratriaconta-19,22,25,28,31-pentaenoic acid (34:5) has been described in Marine Sponge (*Petrosia pellasarca*) [120].

There are numerous modifications of omega-3 fatty acids, and many of them play the role of hormones or compounds produced in stressful situations in plants. We only mention a few examples that occur in dietary sources. The most important seem to be the previously mentioned 2-hydroxylinolenic acid and two hydroxy acids occurring in plants of the genus *Lesquerella—*densipolic and auricolic acid [130,163,164,165,166]. One of them, densipolic acid, was found in *Linum usitatissimum* [132]. Another example is (9Z)-12-hydroxy-9-dodecenoic acid (HDA), which is one of the main products of the lipoxygenase pathway, widespread among plants [126,127,128]. Additionally, oxo fatty acids are prevalent in the plant world. For instance, 12-oxo-*cis*-9-dodecenoic acid is one of the reaction products catalyzed by the fatty acid hydroperoxide lyase, present in many plants, in large quantities, inter alia, in mature soybeans [124,125]. Table 1 lists the acids belonging to all of the groups discussed above.

## 3. Bioavailability

Bioavailability is a relative term, which can refer to both the speed of absorption and the quantity of the substance absorbed. The speed can be understood as the rate at which the substance is absorbed from the gastrointestinal tract and reaches the portal system. Absorption of the substance occurs in the gastrointestinal tract only to a certain extent, depending on many factors. The extent of absorption and the speed of substance transport to the portal circulation describe the bioavailability in the narrower sense. Traditionally, bioavailability can also be considered in a broader context, taking into account the amount of substance that reaches the systemic circulation or the place of physiological destiny (activity) [167]. This broader approach is particularly important when considering the effect of metabolic processes and excretion on the transport of substances from the portal circulation. Not all of the absorbed substance reaches the systemic circulation or tissue compartment consistent with the physiological destination. This difference in amount is very important from the point of view of pharmacokinetics and dietary planning. 

Fatty acids may be present in the body as free fatty acids, bound to glycerol, to form triacylglycerol (TG), diacylglycerol (DAG) or monoacylglycerol (MAG), or to form a composition of membrane phospholipids. In naturally occurring TG molecules, LC PUFA occupies the second position [167]. In the phospholipids of cell membranes the latter position is competed by EPA and DHA with arachidonic acid, and if necessary, they are released by the enzyme phospholipase A2 and are used to synthesise eicosanoids [136]. Otherwise, in the human brain, VLCFAs (C34–38) are attached to the skeleton of glycerol (glycerol moiety), which in phospholipids are located in the *sn*-1 position [122].

In fish and fish oils, LC omega-3 PUFAs are mainly found as triacylglycerides and free fatty acids [167,168]. In Krill oil phospholipids are also an important fraction of these fatty acids (30–65% of EPA and DHA), mainly phosphatidylcholine [167,169,170,171]. EPA and DHA represent approximately 18% and 12%, respectively, of the content of naturally occurring fish oils [167]. However, due to the transesterification process, oil blends often contain much more of both EPA and DHA. This process is related to the substitution of the removed glycerol backbone with ethanol, resulting in the formation of ethylesters (EE), which can then be converted to re-esterified TG (rTG)-ethanol is enzymatically removed, resulting in free fatty acids being released, then attached by enzymes back to the glycerol backbone [167,168]. An example of a drug in which the content of EPA (DHA) is increased as a result of transesterification is Lovaza [168,172]. Another method to increase the content of EPA and DHA has been used in Epanova. Glycerol is removed and replaced with a hydrogen atom, which, in combination with the released fatty acid, forms a carboxylic acid. Ethylesters (of which Lovaza is composed) require the hydrolysis of the ester bond by pancreatic lipase before they release the free fatty acids that can be absorbed in the small intestine. This step is not required by the carboxylic acids. Interestingly, EPA and DHA-EE are also absorbed unchanged. However, this form accounts for < 1% of the total pool of EPA and DHA in circulation after ingestion of omega-3 acids EE [168]. 

In rTG, LC PUFAs take up not mainly the *sn*-2 position (which takes place in natural TG), but can also (simultaneously) be bound in the position of *sn*-1 or *sn*-3 with equal paradigmicity. rTG particles frequently contain two LC PUFAs—then the probability of binding EPA and DHA in the *sn*-1 or *sn*-3 position is higher than in the *sn*-2 position [167]. According to Schuchardt, [167] binding of LC omega-3 PUFA to glycerol in the *sn*-1/3 position facilitates the lipase hydrolysis of the bond, thus increasing bioavailability. According to Dyerberg, [173] the presence of MAG and DAG in rTG mixtures increases the absorption of LC PUFAs in the intestine due to the easier formation of micelles. Bandarra [174], in turn, based on the results of his research on hamsters, proves that the location of DHA in the *sn*-2 position increases the absorption of this acid in the intestine and its incorporation into tissues. However, a certain limitation of Bandarra’s study is that the author used a commercially available fish oil, which is known only to be rich in DHA with an unspecified binding site with the glycerol backbone—we do not know what part of DHA is associated in positions other than *sn*-2.

### 3.1. Methods of Measuring the Bioavailability of Omega-3 Fatty Acids

We can measure omega-3 FAs concentration in plasma, serum, blood cells and lymph. The content of FAs in the plasma reflects the short to medium-long supply of fatty acids in the diet, while the concentration of fatty acids in the blood cells is usually a good indicator of long-term bioavailability [167,175]. As far as the long-chain omega-3 fatty acids are concerned, it is possible to measure many markers that indicate the presence of DHA in a specific form, but only one (the level of phospholipid EPA in plasma) that is useful for determining the level of EPA [167,176]. Admittedly, erythrocyte EPA is a weak dose-dependent indicator of LC omega-3 PUFAs substitution at normal dietary levels, however (sum of), erythrocyte EPA and DHA concentration seems to be, as will be mentioned below, a relatively good indicator of long-term bioavailability and also reflects the content of LC omega-3 PUFAs in non-blood tissues [167,177]. In Browning’s study, [177] EPA + DHA-PC (in the case of sudden changes in intake) and platelet/mononuclear cells EPA + DHA (in the case of long-term consumption assessment) were considered biomarkers that best represent the intake of fish with high fat content in a typical UK population (1–4 servings a week). 

### 3.2. Chemical Binding

The bioavailability of omega-3 fatty acids can also be expressed by calculating different coefficients, the most important of which seem to be omega-3 index, C_max_ and AUC_t_ (area under the (concentration-time) curve), the modification of which is the incremental area under the curve (iAUC_t_) [169]. 

The omega-3 index is defined as the proportion of the sum of EPA and DHA content in the total fatty acid content in the erythrocyte (erythrocyte membrane), and is expressed as a percentage [167,178,179]. It is a good indicator of long-term bioavailability (from the last 80–120 days [179]) due to the long lifetime of erythrocytes and their high number in the blood. Plasma content of EPA and DHA, as well as many other fatty acids, weakly correlates or does not correlate at all with the levels of these acids in the erythrocyte membrane [179]. The omega-3 index is also a good indicator of the incorporation of fatty acids into tissues, and this applies not only to gastrointestinal tissues, but also to the myocardium, liver, and kidney [167,180]. It was established that the supply of long-chain omega-3 fatty acids at the level of 1 g/d may result in an increase in the omega-3 index by two percentage points over eight weeks [181]. The omega-3 index is influenced by many factors, such as smoking, physical activity (the intensity of which decreases the omega-3 index), and genes [178]. 

The maximum levels of EPA and DHA in plasma (C_max_) can be determined five–nine h after administration, while the persistent levels of EPA and DHA in plasma are achieved within two weeks of daily supplementation. The half-life of EPA and DHA after repeated administration is 37 h and 48 h, respectively [170].

The bioavailability of omega-3 fatty acids varies depending on the type of chemical binding (lipid structure), and can be ranked as follows: PL > rTG > TG > FFA > EE [167,178,179,182,183]. Both EE and rTG are not natural components of dietary oils, but they are created in the process of their chemical modification, which is called transesterification. As a result, highly concentrated oils containing up to 90% of EPA and DHA can be obtained [167]. The hierarchical order presented above does not, however, reflect reality to the extent that some authors would like it to. In their trial Kohler et al. [178] proved that the bioavailability of EPA and DHA in the form of phospholipids does not have to be greater than the bioavailability of these acids in the form of triglycerides—the bioavailability of EPA and DHA in krill meal was comparable to their bioavailability in fish oil, despite a slightly higher total fat content in krill meal. Yurko-Mauro et al. [184] achieved similar results—krill oil containing almost 44% of phospholipids showed bioavailability similar to the bioavailability of fish oil, both in the form of triglycerides and ethyl esters. This indicates the (great) share of non-chemical binding (and total fat content) factors in shaping the bioavailability of omega-3 fatty acids [169,178,185]. One explanation may be the observation of Nordoy, Reference [186], who noticed similarly good absorption of omega-3 fatty acids (including EPA and DHA) in the form of TG and EE if they are administered as a component of fish oil in equivalent amounts. Both krill oil, and to a lesser extent, krill meal and fish oil, which are rich in fat. 

Many studies, however, maintain that krill oil, especially when very rich in phospholipids, is characterized by extremely high bioavailability, significantly higher than triglyceride-rich fish oil [170,187,188,189]. Those also find an understandable explanation. Between the omega-3 PUFAs bound to phospholipids and those linked to triglycerides, there is some difference associated with predestination to a specific type of blood transport and metabolism in the liver. DHA-TG is preferentially assigned to LDL-PL, while DHA-PL is preferentially assigned to HDL-PL. It was also found that omega-3 PUFAs, including DHA, in the form associated with phospholipids, are more intensely embedded in tissues. This is probably due to the better availability of LC *n*-3 PUFA-PL acids contained in krill oil than those present in fish oil LC *n*-3 PUFA-TG for liver beta-oxidation pathways [189]. Krill oil inhibits de novo lipogenesis, but enhances fatty oxidation [170].

### 3.3. Brain Transport

Omega-3 acids are incorporated into the cell membrane of many organs and tissues, above all the heart, nervous tissue and retina [167]. Oral supplementation with omega-3 PUFAs increases the content of these acids in the cerebrospinal fluid [190]. Efficient passage of the blood-brain barrier, however, requires carrier particles—in the case of DHA, it is 1-lyso, 2-docosahexaenoyl-glycerophosphocholine (LysoPC-DHA), which increases intracerebral DHA transport up to 10-fold. It is a brain-specific particle and does not facilitate the transport of DHA to the heart, liver or kidney, although detailed studies are required in humans. Carriers (transporting DHA to the brain) with potentially better properties are synthesized, an example of which is obtaining of AceDoPC (1-acetyl,2-docosahexaenoyl-glycerophosphocholine) [191]. 

### 3.4. Parenteral Administration

Most of the studies, especially those based on humans, which serve to determine the bioavailability of omega-3 acids, apply to their oral administration. It is difficult to fully validate the parenteral administration of omega-3 fatty acids in relation to the healthy population, because this method of supply is reserved mainly for patients undergoing intensive therapy, both adults and preterm infants [142,192,193]. In addition, it is worth noting that parenteral administration of mixtures based on fish oil may lead to biochemical liver damage and even the progression of fibrosis in this organ [194].

Al-Taan et al. [195] conducted a study on 20 patients awaiting the surgical removal of colorectal metastases from liver cancer. These individuals had normal liver function tests and plasma lipid levels within the reference range. The aim of the study was to assess the content of fatty acids in plasma phosphatidylcholine and erythrocytes during and after intravenous infusion of oil emulsion. Phosphatidylcholine (PC) is the main phospholipid that can be found in the circulation (blood) and erythrocyte (membrane) during and shortly after intravenous infusion of the oil emulsion. Parenteral administration of DHA and EPA lipid emulsion allowed a rapid and significant increase in their blood levels (EPA/DHA in plasma PC and EPA in erythrocytes). However, EPA levels returned to their initial values five–12 days after the end of the infusion. Not only Al-Taan, but also Browning [177], suggested a quicker turnover of EPA than DHA in cells, and thus this effect occurs with both oral and intravenous supply, with a different time of administration. The fact of a relatively short infusion of oil emulsion is also significant.

### 3.5. Matrix Effect and Emulsification

Many authors emphasize the key role of the fat content in food in the absorption of omega-3 acids (‘the matrix effect’ [167,179]) and suggest the necessity of introducing a recommendation to consume formulations containing omega-3 fatty acids with high-fat food [167,178,196]. Schuchardt underscores the lack of the expected cardioprotective effect in the German population supplementing omega-3 PUFAs during breakfast due to the relatively low-fat typical German breakfast [167]. Similarly, American society is in the habit of eating a low-fat breakfast, which in their case contains only 16% of the fat consumed during the day [197]. In addition, the fat content of food is sometimes inversely proportional to the amount of omega-3 fatty acids it contains. For example, high-fat plants may have few omega-3 fatty acids—*Entandrophragma angolense—*a potential health benefit food source has fat estimated as 59.43% of fresh weight. However, 33.29 weight % of *Entandrophragma* seed oil contains oleic acid and only 0.2 weight % is *alpha*-linolenic acid [139,198]. Chia, on the other hand, contains oil in the amount of 27 g per 100 g of seeds, of which 64.04% is ALA and only 14.98% are saturated fatty acids [68]. Soybean contains slightly less fat. However, the high content of linoleic acid and the unfavourable omega-6/omega-3 ratio make it a food of dubious health value, especially because it has been shown that induction of obesity in mice with soybean oil is possible [95,139,199].

The fat contained in the diet stimulates the pancreas to secrete fat-digesting enzymes and the gallbladder to eject bile that contains bile salts, which emulsify fats and activate pancreatic lipase [196]. However, it is recommended that persons with a high cardiovascular risk should reduce the supply of animal fat. On the other hand, substituting animal fat with vegetable fat is not necessarily a good solution, taking into account eating habits, ubiquitous overweight and often low content of omega-3 fatty acids in rich-fat plants. These people benefit from the achievements of nanotechnology, among which Cavazos-Garduno [200] mentions nanoparticles, nanospheres, nanocapsules, solid lipid nanoparticles (SLN), self-emulsifying drug delivery systems (SEDDS) and nanoemulsions, which significantly improve the absorption of fatty acids in a low-fat environment. Research indicates that particles smaller than 0.2 micrometers are absorbed better. 

The self-microemulsifying delivery system (SMEDS) contains LC *n*-3 FA-EE and a number of compounds that are emulsified in the stomach without the need for a high-fat meal [196]. It has been known for many years that emulsified omega-3 acids are characterized by equally good bioavailability in poor and high fat environments [196,201]. Qin analysis [196] indicates that the absorption of DHA + EPA EE contained in SMEDS preparations is 6 times higher than DHA + EPA EE alone. To confirm the effectiveness of SMEDS technology, long-term studies are needed to analyze the embedding process of the above-mentioned fatty acids in the structure of cell membranes, especially erythrocytes.

The benefits of emulsification were also described by Puri, [138]. He used similar self-nanoemulsifying drug delivery systems (SNEDDS).

Absorption of omega-3 fatty acid ethylesters can also be enhanced through the use of Advanced Lipid Technologies (ALT). ALT is, according to the manufacturer, a special lipophilic system that, regardless of the supply of food and the fat content thereof, increases the bioavailability of lipid-based compounds, including omega-3 fatty acid ethyl esters, generating the spontaneous formation of micelles. An example of a preparation equipped with this component is SC401 (DHA and EPA ethylesters + Advanced Lipid Technologies). In the supply of low-fat food, the intake of SC401 was associated with significantly higher values demonstrating the bioavailability of DHA and EPA than Lovaza (nearly 2-fold higher C_max_ and 3-fold higher AUC_(0-last)_). 

Free fatty acids are another example of a formula well absorbed with a low fat content in the diet. Epanova bioavailability in conditions of low fat supply is much greater than with Lovaza (AUC_(0−t)_ for Epanova is four times higher than for Lovaza), which in turn, consumed with high-fat food, has similar bioavailability as SC401 accepted in conditions of low food supply [201]. 

Vegetarians and vegans will also be able to use the emulsification technique in line with their own lifestyle, as the results of the latest research indicate the possibility of using vegetable proteins from pea, lentil, and faba bean as emulsifiers [202]. Moreover, oil-in-water nanoemulsions are constructed using vegetarian oils (algae and flaxseed oil) [202,203].

A very common method of the incorporation of nanoemulsion into food is its transformation, most often into the form of powder, using microencapsulation [204]. Microencapsulation stabilizes the oil mixture, protects it against oxidation, and eliminates the phase difference often present when mixed with food [181]. Microencapsulation of fish oil increases the bioavailability of omega-3 PUFAs to a value very close to the bioavailability of these acids in meals rich in fish oil in liquid form [205]. Emulsification increases the bioavailability of LC omega-3 PUFAs by increasing the efficiency of incorporation into triacylgliceryde-rich lipoproteins [206]. Emulsifiers also modify the expression of genes responsible for the transport of fatty acids in enterocytes [143]. Emulsification (pre-emulsification) increases the absorption of LC PUFAs, including DHA, EPA, and ALA, but does not have the effect on the absorption of fatty acids having shorter chain length and fewer unsaturated bonds [143,207,208]. 

Of course, emulsification elevates not only the bioavailability of omega-3 acids in the blood, but also in the lymph. Emulsifiers used in food are part of this mechanism. For example, soya lecithin added to flaxseed oil increases the number and size of ALA-rich chylomicrons produced by enterocytes. However, another emulsifier, sodium caseinate, significantly reduces the absorption of ALA in the intestine. This is probably related to the effect on the expression of the FABP2 gene, which is involved in the transport of fatty acids in enterocytes. It was noted that the presence of soy lecithin in the emulsion was accompanied by high expression of this gene, whereas the use of sodium caseinate was associated with a decrease in FABP2 gene expression [143]. This process requires careful research on humans, because it is likely that the effect of emulsifiers on gene expression should be considered during food production, especially when we expect a specific healing effect. 

## 4. Interactions between Long-Chain Polyunsaturated Fatty Acids

Linoleic acid (LA), which is omega-6 fatty acid, competes with ALA for enzymes converting *alpha*-linolenic acid to EPA, DPA, and DHA. LA is (similarly to arachidonic acid), inter alia, the precursor of eicozanoids, while DHA, DPA, and EPA can be transformed into anti-inflammatory agents. [25,26,209]. Changes in the supply of omega-6 PUFAs affect the plasma concentration of omega-3 fatty acids. This applies to both non-esterified omega-3 fatty acids and those associated with phospholipids, triglycerides, and cholesteryl esters. Taha et al. [209] showed that reducing the supply of omega-6 PUFAs for 12 weeks increases the concentration of omega-3 PUFAs in the plasma. In addition, the simultaneous increase in the supply of omega-3 PUFAs for a dozen weeks further elevates the concentration of these acids in the plasma and lowers the level of arachidonic acid. An important interaction of omega-6 PUFAs with omega-3 PUFAs is the production of non-native thromboxanes and leukotrienes [170]. This probably results, among others, from the substrate competition for the catalytic center of desaturases and elongases, and the reduction of arachidonic acid production with a sufficiently high supply of *n*-3 PUFAs.

Based on Wood’s critical analysis, [185], it can be concluded that an increase in EPA level and also in a certain degree of DHA can probably be achieved by reducing the supply of linoleic acid and/or increasing the intake of *alpha*-linolenic acid, although the extent of the change is small. It is suggested to maintain the ratio of omega-6/omega-3 consumption in the range from 1/5 to 1/10, and ideally, as in Japanese society, from 1/2 to 1/4 [141,210]. In turn, for breastfeeding mothers, this ratio should not exceed 10 [211]. Meanwhile, the diets of Western societies are characterized by a clear advantage of the supply of omega-6 fatty acids and the ratio of *n*-6/*n*-3 shaping in the range of 15/1 to 16.7/1 [212]. The discussion about the preferred ratio of consumption of omega-6 to omega-3 fatty acids has been going on for many years and still has not produced convincing results. Researchers are wondering about the legitimacy of returning to an “ancestral diet”, which is attributed to the balanced consumption of both groups of fatty acids at the level of 1:1 [213,214,215,216]. Attention is also paid to certain health benefits that may result from a strictly defined omega-6 to omega-3 ratio, as exemplified by the postulated beneficial effect of the 4:1 ratio on the functioning of the nervous system or reduction of tumor cell proliferation in patients with colorectal cancer who they were nourishing themselves, keeping the ratio 2.5:1 in the diet [212,217,218,219]. The above authors indicate the desirability of consuming up to four times more omega-6 fatty acids than omega-3 and this value depends on the disease under consideration. Regardless of the symptoms or lack thereof, most researchers postulated not exceeded intakes omega6/omega-3 at 10 [211,213]. In light of eating habits of the societies of many highly developed countries, a certain compromise seems to be current recommendations for the consumption of omega-6 and omega-3 fatty acids, which shape the omega-6/omega-3 index in the range from 7:1 to 11:1 [220].

Kohler et al. [178] studied the bioavailability of DHA and EPA added in the form of ethyl esters to sausages with similar ALA content. Both the Omega-3 index and the percentage of EPA and DHA in erythrocytes increased significantly more in the verum group than in the control (placebo) group, in which they remained almost unchanged. The content of DPA similarly increased in both groups. The absence of a significant increase in EPA and DHA in the control group, despite ALA supplementation in both groups, is not surprising, as ALA is converted to EPA and DHA to a very small extent [136,140,141]. The difference in the increase in EPA, DHA and DPA levels in the control group may be due to the different conversion efficiency of ALA to the above-mentioned acids, although this is not the only reason, because these acids may also be converted into each other. Interestingly, the ALA level increased slightly in the control group (*p* = 0.038), while in the treatment group it decreased slightly (*p* = 0.141). Despite the small difference in these ALA levels and the lack of statistical significance of ALA decrease in the treatment group, the explanation of large differences in ALA levels after supplementation between individual participants requires careful research on the metabolic interactions of individual omega-3 fatty acids.

## 5. Lipid Oxidation 

LC omega-3 PUFAs are sensitive to oxidation [206,221]. Some studies report increased levels of peroxidized lipids in fish oil capsules, and as their regular consumption may adversely affect health, this problem needs to be considered [206]. One of the ways of protecting omega-3 acids against oxidation is the microencapsulation of oils using WPI-GA complex coacervates and spray dried microcapsules [221]. One of the less expensive alternative methods is ionic gelation using *Salvia hispanica* mucus in combination with sodium alginate and calcium [222]. The effect of oxidation on the bioavailability of omega-3 fatty acids is not clear. According to Staprans and Naruszewicz [223,224], the consumption of peroxidized vegetable oils increases the number of peroxidized lipids in chylomicrons. Ottestad [206], on the other hand, reports that consumption of oxidised cod liver oil has no effect on the incorporation of omega-3 PUFAs into lipoproteins rich in triacylglycerides.

## 6. Conclusions

Over recent years, there has been significant progress in research on the bioavailability of omega-3 fatty acids. We know more and more of their sources and we understand better the importance of their chemical structure and the form of supplementation. People consume omega-3 fatty acids mainly with fish, and to a lesser extent with cereal products and meat. Some of them, however, do not appear in the abovementioned products, or their presence is unexplored. In addition, the content of individual acids in plants depends on the place of their occurrence, and in animals on the type of diet. Despite the high availability of seafood, which is rich in many omega-3 acids, the authors recognize the need to constantly search for new sources of omega-3 fatty acids due to the risk of excessive exposure to mercury methyl and other toxic compounds present in fishing areas. In addition, some acids, such as coniferonic or juniperonic acid, occur mainly in plants that are not widely used in consumption, or are even poisonous, but can be used in the pharmaceutical industry and medicinal chemistry. This is important because of the many reasons for finding omega-3 fatty acids and their derivatives, valuable active substances that may be important in the development of new drugs and therapeutic regimens. Opinions about the bioavailability of omega-3 fatty acids are divided. Some believe that phospholipid-bound acids are absorbed better, as well as more intensely incorporated into tissues than those associated in triglyceride form, due to the specificity of blood transport and better accessibility of beta-oxidation pathways. Others, however, are of the opinion that factors unrelated to the type of chemical binding play a key role, and the fat content in food is decisive. In addition, promising effects seem to result from the use of special lipophilic systems and nano(micro)emulsions. However, the type of emulsifier used should be taken into account, as some influence the expression of the gene involved in the transport of fatty acids in enterocytes in various ways. Fatty acids affect each other’s metabolism in the body, which is of dietary importance. Several times higher consumption of omega-6 than omega-3 fatty acids is recommended by most researchers. Omega-3 fatty acids, like all fatty acids, are highly susceptible to oxidation. There are methods that disrupt this process and may be used in the production of supplements, but the significance of the process itself for bioavailability is unclear. 

It is hoped that reading the above review encourages scientists to carry out further research, which will dispel these and many other doubts.

## Figures and Tables

**Table 1:** Omega-3 fatty acids—chemistry and sources.

No	IUPAC Name	Common Name	Shorthand (Simplified Formula)	Molecular Formula	Formula/Structure	Molecular Weight	Sources [Refs.]
**MONOUNSATURATED FATTY ACIDS (MUFAs)**							
1	(9*Z*)-dodec-9-enoic acid	9-lauroleic acid/lauroleic acid	C12:1*n*-3	C_12_H_22_O_2_	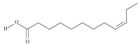	198.306 g/mol	0.08% of fatty acid composition (total free fatty acids) of fresh and mature cheese samples of commercial (with full fat content) Manchego-type cheese and 0.05%/0.07% of the same cheese samples (fresh and mature) made from low-fat/full-fat milk with the addition of 2 g avocado oil per 100 mL of milk [35]; 0.31% of free fatty acids from bovine whey cream [36]
2	(9*E*)-dodec-9-enoic acid	-	C12:1*n*-3	C_12_H_22_O_2_	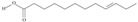	198.306 g/mol	in *Peganum harmala* fatty acids (concentration 0.31%) [37]
3	(11*Z*)-tetradec-11-enoic acid	-	C14:1*n*-3	C_14_H_26_O_2_	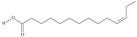	226.36 g/mol	a precursor of the sex pheromone component in *Ostrinia scapulalis* [38]
4	(11*E*)-tetradec-11-enoic acid	-	C14:1*n*-3	C_14_H_26_O_2_	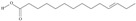	226.36 g/mol	13.37% of leaf essential oil of *Coriandrum sativum* [39]; in *Spodoptera littoralis* [40]
5	(13*E*/*Z*)-hexadec-13-enoic acid	-	C16:1*n*-3	C_16_H_30_O_2_	undefined *cis*-*trans* isomerism	254.414 g/mol	in microalgae—1.39%, 3.12%, 0.23%, 1.33% of *Dunaliella tertiolecta*, *Platimonas viridis*, *Nefrochloris salina* and *Phaeodactylum tricornutum* total fatty acids, respectively, also present in the gonads and larvae of oysters fed with the mentioned microalgae [41]
6	(13*E*)-hexadec-13-enoic acid	-	C16:1*n*-3	C_16_H_30_O_2_	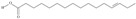	254.414 g/mol	in *Brassica napus* leaf discs—3.35%, 1.06%, 1.19% of total PG (phosphatidylglycerol), PA (phosphatidic acid) and PC (phosphatidylcholine) fatty acids, respectively, trace amounts in phosphatidylethanolamine (PE) were also found [42]; in the moth *Spodoptera littoralis* [43]
7	(13*Z*)-hexadec-13-enoic acid	-	C16:1*n*-3	C_16_H_30_O_2_	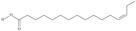	254.414 g/mol	in microalga *Heterosigma carterae* [44]
8	(15*E*/*Z*)-octadec-15-enoic acid	-	C18:1*n*-3	C_18_H_34_O_2_	undefined *cis*-*trans* isomerism	282.468 g/mol	detected in pork (4.2 mg/100 g fatty acids of the fresh ham of adult pig, 4.8 mg/100 g fatty acids of the pork loin of adult pig) [45])
9	(15*Z*)-octadec-15-enoic acid	-	C18:1*n*-3	C_18_H_34_O_2_	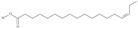	282.468 g/mol	detected in beef (0.217% (for the grass-fed beef samples) and 0.241% (for conventional beef) of total fatty acids mass) [46], cows milk (0.146–0.56 g/100 g of total fatty acids, dependently on applied diet) and butter [47], and in human blood (relative abundance for healthy male—0.04%) [48] and human milk (0.01–0.06% of total fatty acids of transitional and mature human milk from five regions in China [49], 0.02–0.29% by weight of total fatty acids in human milk samples collected from woman in the United States {the mean concetration for both (*cis* and *trans*) isomers—0.06%} [50])
10	(15*E*)-octadec-15-enoic acid	-	C18:1*n*-3	C_18_H_34_O_2_	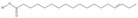	282.468 g/mol	detected in cow’s milk (0.102–0.34 g/100 g of total fatty acids, dependently on applied diet) and butter [47], 0–0.15% by weight of total fatty acids in human milk samples collected from women in the United States (the mean concetration for both (*cis* and *trans*) isomers—0.06%) [50]
**POLYUNSATURATED FATTY ACIDS (PUFAs)**							
1	(11*Z*,13*Z*)-hexadeca-11,13-dienoic acid	-	C16:1*n*-3	C_16_H_28_O_2_	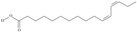	252.398 g/mol	in the (pheromone) gland extracts of the navel orangeworm, *Amyelois transitella* [51]
2	(7*Z*,10*Z*,13*Z*)-hexadeca-7,10,13-trienoic acid	roughanic acid	C16:3*n*-3	C_16_H_26_O_2_	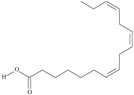	250.382 g/mol	as methyl ester: 0.11–0.12% of European wels catfish oil, 0.34–0.41% of common bleak oil (in Area (%)) [52]; 3, 1.1, 0.9, 6.6, 0.4, 4.1, 0.5, 0.5, 0.3, 0.1, 11.8% of Chinese cabbage, white cabbage, savoy, kale, red cabbage, brussels sprouts, cauliflower, kohlrabi, swede, cabbage lettuce, parsley fatty acid mass [53]—systematic names were placed in the line ‘*α*-linolenic acid’; in leaves of many conifer species, most in *Larix leptolepus* and *Taxus baccata*—4.8% and 5.8% of fatty acids of lives, respectively [54]
3	(4*Z*,7*Z*,10*Z*,13*Z*)-hexadeca-4,7,10,13-tetraenoic acid	-	C16:4*n*-3	C_16_H_24_O_2_	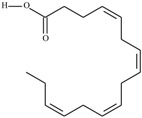	248.366 g/mol	as methyl 4,7,10,13-hexadecatetraenoate: 0.09–0.1% of Pontic shad oil, 0.08–0.16% of European wels catfish oil, 0.11–0.56% of common bleak oil [52]
4	(8*Z*,11*Z*,14*Z*)-heptadeca-8,11,14-trienoic acid	norlinolenic acid	C17:3*n*-3	C_17_H_28_O_2_	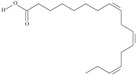	264.409 g/mol	in *Salvia nilotica*—0.4% of mixed esters and 2.3% of IV fraction (% by gas liquid chromatography) [55]
5	(9*Z*,12*Z*,15*Z*)-octadeca-9,12,15-trienoic acid	*α*-linolenic acid	C18:3*n*-3	C_18_H_30_O_2_	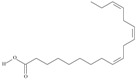	278.436 g/mol	0.2% of black cumin (*Nigella sativa*) seed oil fatty acids [56]; 8% of *Cardiospermum halicabum* seed oil total fatty acids [57]; as methyl esters: 1.67% of *Labeo rohita* muscle tissue fatty acids, 1.39% of *Cirrhinus mrigata* muscle tissue fatty acids, 1.29% of *Catla catla* muscle tissue fatty acids [58]; in salted products of fish roe: 0.7% of Ikura (salmon), 0.4% of Tarako (pollock), 0.7% of Tobiko (flyingfish), 0.4% of Kazunoko (herring) total lipids [59]; 1.6–1.9% of salmon eggs total phospholipid fraction, 1–3.6% of salmon eggs total triacylglicerol fraction [60]; 1.1% of wild sardine (*Sardina pilchardus*) muscle total fatty acids (1% of captive sardine muscle total fatty acids), 0.7% of wild/captive sardine liver total fatty acids [61]; 0.61–0.83% for finwhale oils, 0.13–0.34% for seal oils [62]; 0.5% of tuna oil [63]; 0.5% of *Epinephelus fasciatus* muscle triacylglycerols, 0.1% of *E. fasciatus* PE and 0.1–0.2% of *E. fasciatus* PC, 0.1–0.5% of *E. retouti* muscle triacylglycerols, 0–0.1% of *E. retouti* PE and 0.1% of *E. retouti* PC [64]; 16.4 g/100 g of the milled chia seeds [65]; 21% of n-hexane extract of *Senna italica* aerial parts [66]; 40% of *Chamaecyparis lawsoniana* and 50% of *Fokienia hodginsii* total fatty acids [67]; 64.04% of chia (*Salvia hispanica*) seed oil fatty acids [68]; 0.25 g/100 g of total fatty acid methyl esters of milk fat from ewes, 2.57 g/100 g of total fatty acid methyl esters of milk fat from ewes fed diets with extruded linseed supplementation (12% linseed in dry matter) [69]; 59.5, 57, 46.4, 50, 39.8, 58.7, 48.9, 41.9, 50.8, 54.6, 41.7, 10, 6, 8.5, 5.9% of Chinese cabbage (*Brassica chinensis*), white cabbage (*Brassica oleracea*), savoy (*Brassica oleracea* var. *sabauda*), kale (*Brassica oleracea* convar. *acephala* var. *sabellica*), red cabbage (*Brassica oleracea* var. *capitata*), brussels sprouts (*Brassica oleracea* var. *gemmifera*), cauliflower (*Brassica oleracea* convar. *botrytis* var. *botrytis*), kohlrabi (*Brassica oleracea* convar. *acephala* var. *gongylodes*), swede (*Brassica napus* var. *napobrassica*), cabbage lettuce (*Lactuca sativa* var. *capitata*), parsley (*Petroselinum crispum* ssp. *crispum*), black salsify (*Scorzonera hispanica*), carrot (*Daucus carota* ssp. *sativus*), turnip-rooted parsley (*Petroselinum crispum* ssp. *tuberosum*), Florence fennel (*Foeniculum vulgare* var. *azoricum*) fatty acids mass, respectively; 8.7, 9.7, 11.5, 8.3, 10.2, 11.2, 9.3, 11, 12.3% of Chinese cabbage, white cabbage, savoy, kale, red cabbage, brussels sprouts, cauliflower, kohlrabi, swede, black salsify, cabbage lettuce, carrot, parsley, Florence fennel seed fatty acids mass, respectively [53]; in *Trichosanthes kirilowii* (33.77–38.66% of seed oils) [70]; in *Linum usitatissimum* (depending on the genotype it contains from 1.1 to 65.2% of total fatty acids in seeds) [71]; in paprika *Capsicum annuum* (29.93% of fresh pericarp fatty acids in Jaranda variety and 30.27% in Jariza variety) [72]
6	(9*E*,12*E*,15*E*)-octadeca-9,12,15-trienoic acid	linolenelaidic acid/elaidolinolenic acid	C18:3*n*-3	C_18_H_30_O_2_	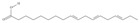	278.436 g/mol	in Turkish sage species—*Salvia virgata*, *Salvia potentillifolia*, *Salvia recognita*, *Salvia tomentosa* amounts to 0.4, 0.7, 1.1 and 1.4% of seed fatty acids, respectively [73]; 0.03% of tobacco seed oil fatty acids [74]
7	(9*Z*,11*E*,15*Z*)-octadeca-9,11,15-trienoic acid	rumelenic acid	C18:3*n*-3	C_18_H_30_O_2_	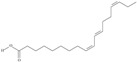	278.436 g/mol	in muscle samples from linseed oil-fed lambs [75]; 0.03 g/100 g of total fatty acid methyl esters of milk fat from ewes, 0.52 g/100 g of total fatty acid methyl esters of milk fat from ewes fed diets with extruded linseed supplementation (12% linseed in dry matter) [69]
8	(9*Z*,11*E*,15*E*)-octadeca-9,11,15-trienoic acid	-	C18:3*n*-3	C_18_H_30_O_2_	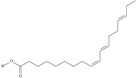	278.436 g/mol	0.01 g/100 g of total fatty acid methyl esters of milk fat from ewes, 0.21 g/100 g of total fatty acid methyl esters of milk fat from ewes fed diets with extruded linseed supplementation (12% linseed in dry matter) [69]
9	(5*Z*,9*Z*,12*Z*,15*Z*)-octadeca-5,9,12,15-tetraenoic acid	Coniferonic acid	C18:4*n*-3	C_18_H_28_O_2_	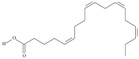	276.42 g/mol	found in many conifer species: 0.05% of *Abies fraseri* seed total fatty acids, 0.03% of A. *nordmanniana* ssp. *nordmanniana* seed total fatty acids, 0.05% of *A. numidica* seed total fatty acids, 0.06% of *A. procera* seed total fatty acids, 0.05% of *Tsuga chinensis* seed total fatty acids, 0.09% of *Tsuga heterophylla* seed total fatty acids, 1.78% of *Pseudolarix amabilis* seed total fatty acids [76]; 2% of *Chamaecyparis lawsoniana* and 2.8% of *Fokienia hodginsii* seed total fatty acids [67]; 11% of *Abies vejarii* Martínez leaf fatty acids [77]
10	(6*Z*,9*Z*,12Z,15*Z*)-octadeca-6,9,12,15-tetraenoic acid	stearidonic acid (SDA)/moroctic acid	C18:4*n*-3	C_18_H_28_O_2_	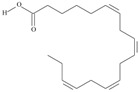	276.42 g/mol	in salted products of fish roe: 0.8% of Ikura (salmon), 0.8% of Tarako (pollock), 0.4% of Tobiko (flyingfish), 0.4% of Kazunoko (herring) total lipids [59]; 2.3% of wild sardine (*Sardina pilchardus*) muscle total fatty acids (1.5% of captive sardine muscle total fatty acids), 0.7% of wild/0.6% of captive sardine liver total fatty acids [61]; as methyl ester: 0.57–0.76% of common barbel oil, 1.52–2.04% of Pontic shad oil, 0.2–0.35% of European wels catfish oil, 0.28–0.59% of common bleak oil [52]; 0.41–0.71% for finwhale oils, 0.79–1.09% for seal oils (in weight per cent) [62]; 0–0.9% of *Epinephelus retouti* muscle triacylglycerols [64]; mean amounts of seeds total fatty acid methyl esters: 4.73% for *Primulaceae*, 4.99% for *Boraginaceae*, 0.36% for *Hippophae rhamnoides*, 0.77% for *Cannabis sativa* [78]; in *Ribes nigrum* and fish oils [77]; 14% and 16.2% of *Echium humile* ssp. *pycnanthum* seeds fatty acids, depending on the location [79]
11	(all-*E*/*Z*)-octadeca-9,11,13,15-tetraenoic acid	parinaric acid	C18:4*n*-3	C_18_H_28_O_2_	undefined *cis*-*trans* isomerism	276.42 g/mol	11.34% of the methanol extract of *Spirogyra rhizoides* [80]
12	(9*Z*,11*E*,13*E*,15*Z*)-octadeca-9,11,13,15-tetraenoic acid	*α*-parinaric acid (*cis*-parinaric acid)	C18:4*n*-3	C_18_H_28_O_2_	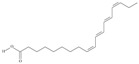	276.42 g/mol	62% of *Parinarium laurinum* seed oil methyl esters [81]; 53.5% of *Parinarium laurinum* seed oil fatty acids [66]; 4% of *Parinarium macrophyllum* seed oil [82]; in *Impatiens edgeworthii* (48% of total fatty acids) [83]; in *Impatiens glandulifera* (42.5% of *Impatiens* oil) [84]; in the seed oil of *Sebastiana brasiliensis* (21.4, 32.5 and 35.1 peak area % of samples from National Park of Turvo, Santana da Boa Vista region and Cacapava do Sul region, respectively) [85]; 30% of *Impatiens balsamina* seed oil [86]; in *Chrysobalanus icaco* (10% of seed oil methyl esters) [81]; in evening primrose (*Oenothera biennis*) oil (3.5 × 10^−5^ mol/L) [87]; in stripped Borage (*Borago officinalis*) oil (1.6 × 10^−4^ mol/L) [82]
13	(9*E*,11*E*,13*E*,15*E*)-octadeca-9,11,13,15-tetraenoic acid	*trans*-parinaric acid (*β*-parinaric acid)	C18:4*n*-3	C_18_H_28_O_2_	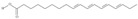	276.42 g/mol	“naturally occurring” [88]; in *Impatiens* spp. [89]
14	(9*Z*,15*Z*)-octadeca-9,15-dienoic acid	Mangiferic acid	C18:2*n*-3	C_18_H_32_O_2_	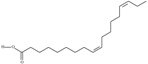	280.452 g/mol	3.1–5.4% of fatty acids in pulp of mango fruit, 1% of mango peel total fatty acids (*Mangifera indica* L.) [90]; in muscle samples from linseed oil-fed lambs [75]
15	(all-*E*/*Z*)-octadeca-11,15-dienoic acid	-	C18:2*n*-3	C_18_H_32_O_2_	undefined *cis*-*trans* isomerism	280.452 g/mol	in beef and mutton tallow—11*E*, 15*E*; 11*Z*(*E*), 15*E*(*Z*); 11*Z*, 15*Z* isomers [91]; 11*E*, 15*Z* isomer in muscle samples from linseed oil-fed lambs [75]
16	(12*Z*,15*Z*)-octadeca-12,15-dienoic acid	-	C18:2*n*-3	C_18_H_32_O_2_	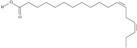	280.452 g/mol	in muscle samples from linseed oil-fed lambs [75]
17	(all-*E*/*Z*)-octadeca-10,15-dienoic acid	-	C18:2*n*-3	C_18_H_32_O_2_	undefined *cis*-*trans* isomerism	280.452 g/mol	in beef and mutton tallow—10*Z*, 15*Z*; 10Z(*E*), 15*E*(*Z*) isomers [92]
18	(11*Z*,14*Z*,17*Z*)-icosa-11,14,17-trienoic acid	eicosatrienoic acid (ETE)/homo-*alpha*-linolenic acid	20:3*n*-3	C_20_H_34_O_2_	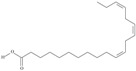	306.49 g/mol	In *Sterculia urens* seeds (2.96% of the total lipid) [91]; in *Hibiscus sabdariffa* seed oil (0.2% of total fatty acids) [93]; 2.22–6.59 mg/g of *Platymonas subcordiformis* dry weight and 24 to 42.04 mg/g of *Porphyridium cruentum* dry weight [94]; 3.97 g/kg soybean unit [95]; 6.78–8.73% of total fatty acids in *Torreya grandis* kernel oil [96]; 31.44% of *Pittosporum undulatum* seed oil total fatty acids [97]; 8.5% of total neutral lipids and 15.5% of free fatty acids of cork *Phellodendron lavalei* seeds [98]; 0.49 g/100g of *Pinus halepensis* seed oil total fatty acids [99]; 0.2% of black cumin (*Nigella sativa*) seed oil fatty acids [56]; 0.01–0.1% for finwhale oils, <0.01–0.03% for seal oils (in weight per cent) [62]; 3.3% of *Ephedra gerardiana* seed lipids and 2.2% of *Cimicifuga racemosa* seed oil [100]; 0.15% and 0.16% of ISA Brown and Arucana hen egg yolk total lipids, respectively [101]; 0.1, 0.2, 0.1, 0.2, 0.2, 0.2, 0.1% of white cabbage, kale, red cabbage, brussels sprouts, cauliflower, cabbage lettuce, parsley fatty acid mass; 0.1, 0.1, 0.1, 0.2, 0.1, 0.2, traces, 0.1, 0.1% of Chinese cabbage, white cabbage, savoy, kale, red cabbage, brussels sprouts, cauliflower, kohlrabi, swede seed fatty acid mass [53]—systematic names were placed in the line ‘*α*-linolenic acid’
19	(8*Z*,11*Z*,14*Z*,17*Z*)-icosa-8,11,14,17-tetraenoic acid	eicosatetraenoic acid (ETA)	C20:4*n*-3	C_20_H_32_O_2_	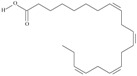	304.474 g/mol	in salted products of fish roe: 2.1% of Ikura (salmon), 0.6% of Tarako (pollock), 0.5% of Tobiko (flyingfish), 0.4% of Kazunoko (herring) total lipids [59]; 2.9–3.8% of salmon eggs total triacylglicerol fraction [48]; as methyl 8,11,14,17-eicosatetraenoate: 0.19–0.24% of common barbel oil, 0.82–1.44% of Pontic shad oil, 0.34–1% of European wels catfish oil, 0.17–0.35% of common bleak oil [52]; 0.35–0.78% for finwhale oils, 0.07–0.26% for seal oils (in weight per cent) [62]; 0.4–0.8% of *Epinephelus retouti* muscle triacylglycerols [64]; 0.06% of Agathis robusta seed lipids [67]; in lipids of bovine liver [102]; in herring (*Clupea harrengus*), mackerel (*Scomber scombus*) and capelin (*Mallotus villosus*) body oils in amounts of 5, 15 and 4 mg/g of fatty acids, respectively, in menhaden (*Brevoortia* spp.) oil and Indian oil sardine (*Sardinella longiceps*)—19 and 6 mg/g of fatty acids, respectively [103]
20	(5*Z*,11*Z*,14*Z*,17*Z*)-icosa-5,11,14,17-tetraenoic acid	juniperonic acid	C20:4*n*-3	C_20_H_32_O_2_	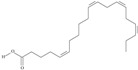	304.474 g/mol	8.9 weight % of *Biota orientalis* seed oil [104]; 0.12–0.57% of *Sargassum* spp. total fatty acids [105]; 1% of *Caltha palustris* seed oil [106]; in many conifer species, most in *Gnetum gnemon*, *Chaemaecyparis thyoides*, *Cephalotaxus sinensis*, *Taxus cuspidata*, *Pseudolarix amabilis*, *Araucaria angustifolia*, *Agathis australis*, *Zamia furfuracea*—12.9, 7.7, 6.8, 6.8, 6.3, 6.3, 6.1, 6, % of leaf fatty acids, respectively [54,77]
21	(5*Z*,8*Z*,11*Z*,14*Z*,17*Z*)-icosa-5,8,11,14,17-pentaenoic acid	eicosapentaenoic acid (EPA)/timnodonic acid	C20:5*n*-3	C_20_H_30_O_2_	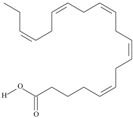	302.458 g/mol	13% of *Undaria pinnatifida* essential oil composition [107]; 3.05 g/100 g (as methyl ester: 3.15% of fatty acids) muscle tissue of *Labeo rohita*, 2.51 g/100 g (as methyl ester: 2.51% of fatty acids) muscle tissue of *Cirrhinus mrigata*, 3.15 g/100 g (as methyl ester: 3.05% of fatty acids) muscle tissue of *Catla catla* [58]; in salted products of fish roe: 13.6% of Ikura (salmon), 18.8% of Tarako (pollock), 7% of Tobiko (flyingfish), 15% of Kazunoko (herring) total lipids [59]; 8.7–10.6% of salmon eggs total phospholipid fraction, 8.3–10.3% of salmon eggs total triacylglicerol fraction [60]; 13.6% of wild sardine (*Sardina pilchardus*) muscle total fatty acids (9.2% of captive sardine muscle total fatty acids), 6.5% of wild/7.2% of captive sardine liver total fatty acids [61]; as methyl ester: 2.32–3.41% of common barbel oil, 2.57–3.8% of Pontic shad oil, 2.41–6.1% of European wels catfish oil, 0.51–1.36% of common bleak oil [52]; 1.82–3.72% for finwhale oils, 6.37–8.12% for seal oils (in weight per cent) [62]; 1.8–3.5% of *Variola louti* muscle triacylglycerols [64]; 2% of *Rhododendron sochadzeae* leaves fatty acids (in hexan extract) [108]
22	(6*Z*,9*Z*,12*Z*,15*Z*,18*Z*)-henicosa-6,9,12,15,18-pentaenoic acid	heneicosapentaenoic acid (HPA)	C21:5*n*-3	C_21_H_32_O_2_	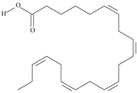	316.485 g/mol	as methyl 6,9,12,15,18-heneicosapentaenoate: 0.06–0.12% of common barbel oil, 0.07–0.08% of Pontic shad oil, 0.09% of European wels catfish oil [52]; 0.2–0.4% of diatom *Skeletonema costatum* total fatty acids, 0.1–0.2% of copepods *Calanus* and *Centropages* sp. total fatty acids, <0.1% of copepods *Temora longicornia* total fatty acids, 0.2–0.7% of euphausid *Meganyctiphanes norvegica* total fatty acids, 0.1–1.1% of euphausid *Euphausia* sp. total fatty acids, 0.1–0.3% of herring oil *Clupea harengus* total fatty acids, 0.19% of sturgeon oil *Acipenser oxyrhynchus* total fatty acids, 0.2–0.6% of mackerel oil *Scomber scrombrus* total fatty acids, 0.1–0.9% of fin whale (*Balaenopterus physalus*) blubber total fatty acids, 0.2% of dolphin (*Tursiops truncatus*) milk total fatty acids [109]
23	(13*Z*,16*Z*,19*Z*)-docosa-13,16,19-trienoic acid	docosatrienoic acid (DTrE)	C22:3*n*-3	C_22_H_38_O_2_	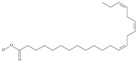	334.544 g/mol	in *Lepidium sativium* seed oil (47.66% of total fatty acids) [110]; trace amount in weed seed oil, largely charlock [111]; in salami-type sausage made of Baltic herring fillets, pork and lard (from < 0.2 to 0.3% during ripening) [112]; in shade dried neem (*Azadirachta indica*) flower powder present to an extent of 5.7% in the total lipid [113]; in eggs (2.92–3.23%), embryos (3.28–3.76%) and larvae (0–3.65%) of *Oncorhynchus mykiss* (from 0 to 3.76% of total fatty acids) [114]; in muscle tissue and liver of *Diplodus vulgaris* (0.8–11.6% of total fatty acids) [115]
24	(7*Z*,10*Z*,13*Z*,16*Z*,19*Z*)-docosa-7,10,13,16,19-pentaenoic acid	docosapentaenoic acid (DPA)/clupanodonic acid	C22:5*n*-3	C_22_H_34_O_2_	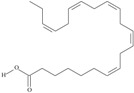	330.512 g/mol	as methyl esters: 1.45% of *Labeo rohita* muscle tissue fatty acids, 1.86% of *Cirrhinus mrigata* muscle tissue fatty acids, 1.09% of *Catla catla* muscle tissue fatty acids [58]; in salted products of fish roe: 5.6% of Ikura (salmon), 1.0% of Tarako (pollock), 2.8% of Tobiko (flyingfish), 1.3% of Kazunoko (herring) total lipids [59]; 6.3–8.1% of salmon eggs total phospholipid fraction, 4.9–6% of salmon eggs total triacylglicerol fraction [60]; 1.6% of wild sardine (*Sardina pilchardus*) muscle total fatty acids (2% of captive sardine muscle total fatty acids), 2.7% of wild/2.4% of captive sardine liver total fatty acids [61]; as methyl ester: 0.6–1.15% of common barbel oil, 0.44–0.83% of Pontic shad oil, 1.04–2.82% of European wels catfish oil, 0.06–0.23% of common bleak oil [52]; 1.07–2.28% for finwhale oils, 2.64–4.74% for seal oils (in weight per cent) [62]; 1.34% of tuna oil [51]; 1.9–3.3% of *Variola louti* muscle triacylglycerols [64]; 13.8/16.7% of wether/eve (lamb) raw meat fatty acids [116]; 0.07% and 1.6% of *Hormosira banksii* and *Dictyota dichomota* total fatty acids, respectively [117]
25	(4*Z*,7*Z*,10*Z*,13*Z*,16*Z*,19*Z*)-docosa-4,7,10,13,16,19-hexaenoic acid	docosahexaenoic acid (DHA)/cervonic acid	C22:6*n*-3	C_22_H_32_O_2_	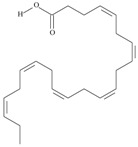	328.496 g/mol	15.44 g/100 g (as methyl ester: 17.98% of fatty acids) muscle tissue of *Labeo rohita*, 18.07 g/100 g (as methyl ester: 8.07% of fatty acids) muscle tissue of *Cirrhinus mrigata*, 17.98 g/100 g (as methyl ester: 15.4% of fatty acids) muscle tissue of *Catla catla* [58]; in salted products of fish roe: 17.4% of Ikura (salmon), 22.2% of Tarako (pollock), 27.9% of Tobiko (flyingfish), 22.6% of Kazunoko (herring) total lipids [59]; 26–29.2% of salmon eggs total phospholipid fraction, 12.5–15.3% of salmon eggs total triacylglicerol fraction [60]; 14.8% of wild sardine (*Sardina pilchardus*) muscle total fatty acids (13.4% of captive sardine muscle total fatty acids), 12.8% of wild/17.1% of captive sardine liver total fatty acids [61]; as methyl ester: 1.36–2.5% of common barbel oil, 4.15–6.47% of Pontic shad oil, 1.44–3.83% of European wels catfish oil, 0.13–0.42% of common bleak oil [52]; 2.74–6.23% for finwhale oils, 5.14–8.22% for seal oils (in weight per cent) [62]; 14.8–19.4% of *Epinephelus fasciatus* musccle triacylglycerols [64]
26	(12*Z*,15*Z*,18*Z*,21*Z*)- tetracosa-12,15,18,21-tetraenoic acid	-	C24:4*n*-3	C_24_H_40_O_2_	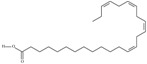	360.582 g/mol	1.3% of Baltic herring (*Clupea harengus*) total fatty acids [118]
27	(9*Z*,12*Z*,15*Z*,18*Z*,21*Z*)-tetracosa-9,12,15,18,21-pentaenoic acid	-	C24:5*n*-3	C_24_H_38_O_2_	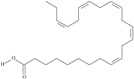	358.566 g/mol	1.4% of Baltic herring total fatty acids [118]
28	(6*Z*,9*Z*,12*Z*,15*Z*,18*Z*,21*Z*)-tetracosa-6,9,12,15,18,21-hexaenoic acid	nisinic acid	C24:6*n*-3	C_24_H_36_O_2_	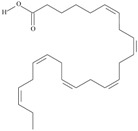	356.55 g/mol	0.9% of Baltic herring total fatty acids [118]
29	(11*Z*,14*Z*,17*Z*,20*Z*,23*Z*)-hexacosa-11,14,17,20,23-pentaenoic acid	-	C26:5*n*-3	C_26_H_42_O_2_	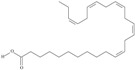	386.62 g/mol	0.5% of of Baltic herring total fatty acids [118]
30	(8*Z*,11*Z*,14*Z*,17*Z*,20*Z*,23*Z*)-hexacosa-8,11,14,17,20,23-hexaenoic acid	-	C26:6*n*-3	C_26_H_40_O_2_	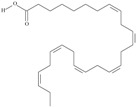	384.604 g/mol	0.7% of Baltic herring total fatty acids [118]
31	(15*Z*,18*Z*,21*Z*,24*Z*,27*Z*)-triaconta-15,18,21,24,27-pentaenoic acid	-	C30:5*n*-3	C_30_H_50_O_2_	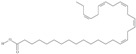	442.728 g/mol	as a methyl ester: 7% of *Cliona celata* total methyl esters [119]
32	(19*Z*,22*Z*,25*Z*,28*Z*,31*Z*)-tetratriaconta-19,22,25,28,31-pentaenoic acid	-	C34:5*n*-3	C_34_H_58_O_2_	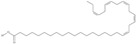	498.836 g/mol	12.4% of the phospholipid fatty acids from *Petrosia pellasarca* [120]
33	(23*Z*,34*Z*)-heptatriaconta-23,34-dienoic acid	-	C37:2*n*-3	C_37_H_70_O_2_	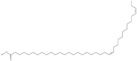	546.965 g/mol	in a CHCl3 extract of Iranian *Rosa x damascena* (*Rosaceae*) [121]
**VERY LONG-CHAIN FATTY ACIDS WITH AN UNDEFINED POSITION OF DOUBLE BONDS**							
34	-	-	C24:4*n*-3	C_24_H_40_O_2_	-	360.582 g/mol	in ram and bull spermatozoa [7]
35	-	-	C24:5*n*-3	C_24_H_38_O_2_	-	358.566 g/mol	in bovine retina [8]; in ram spermatozoa [7]
36	-	-	C24:6*n*-3	C_24_H_36_O_2_	-	356.55 g/mol	in bovine retina [8]; in ram spermatozoa [7]
37	-	-	C26:4*n*-3	C_26_H_44_O_2_	-	388.636 g/mol	in ram spermatozoa [7]
38	-	-	C26:5*n*-3	C_26_H_42_O_2_	-	386.62 g/mol	in ram spermatozoa [7]
39	-	-	C26:5*n*-3	C_26_H_42_O_2_	-	386.62 g/mol	in bovine retina [8]
40	-	-	C26:6*n*-3	C_26_H_40_O_2_	-	384.604 g/mol	in ram spermatozoa [7]
41	-	-	C26:6*n*-3	C_26_H_40_O_2_	-	384.604 g/mol	in bovine retina [8]
42	-	-	C28:5*n*-3	C_28_H_46_O_2_	-	414.674 g/mol	in bovine retina [8]; in ram and bull spermatozoa [7]
43	-	-	C28:6*n*-3	C_28_H_44_O_2_	-	412.658 g/mol	in bovine retina [8]; in ram and bull spermatozoa [7]
44	-	-	C30:5*n*-3	C_30_H_50_O_2_	-	442.728 g/mol	in bovine retina [8]; in ram and bull spermatozoa [7]
45	-	-	C30:6*n*-3	C_30_H_48_O_2_	-	440.712 g/mol	in bovine retina [8]; in ram and bull spermatozoa [7]
46	-	-	C32:5*n*-3	C_32_H_54_O_2_	-	470.782 g/mol	in bovine retina [8]
47	-	-	C32:6*n*-3	C_32_H_52_O_2_	-	468.766 g/mol	in bovine retina [8]—main component of VLC-PUFA-PC of bovine retina [122]; in bull spermatozoa [7]
48	-	-	C32:7*n*-3	C_32_H_50_O_2_	-	466.75 g/mol	in ram and bull spermatozoa [7]
49	-	-	C34:5*n*-3	C_34_H_58_O_2_	-	498.836 g/mol	in bovine retina [8]
50	-	-	C34:6*n*-3	C_34_H_56_O_2_	-	496.82 g/mol	in bovine retina [8]—main component of VLC-PUFA-PC of bovine retina [122]; in ram and bull spermatozoa [7]
51	-	-	C36:5*n*-3	C_36_H_62_O_2_	-	526.89 g/mol	in bovine retina [8]
52	-	-	C36:6*n*-3	C_36_H_60_O_2_	-	524.874 g/mol	in bovine retina [8]
53	-	-	C36:6*n*-3	C_36_H_60_O_2_	-	524.874 g/mol	in normal human brain [122]
**MODIFICATIONS OF OMEGA-3 FATTY ACIDS**							
1	(8*Z*)-5-oxo-11-hydroxyundec-8-enoic acid 11-O-glucoside	-	5-oxo, 11-OH, 11-O-Glu-C11:1*n*-3	C_17_H_28_O_10_	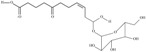	392.40 g/mol	in *Youngia japonica* [123]
2	(9*Z*)-12-oxododec-9-enoic acid	-	12-oxo-C12:1*n*-3	C_12_H_20_O_3_	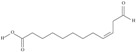	212.289 g/mol	present in many plants in large quantities, inter alia, in mature soybeans [124,125]
3	(9*Z*) -12-hydroxydodec-9-enoic acid	HDA	12-OH-C12:1*n*-3	C_12_H_22_O_3_	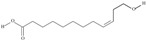	214.305 g/mol	widespread among plants [126,127,128]
4	(9*Z*,11*E*,15*Z*)-13-hydroperoxyoctadeca-9,11,15-trienoic acid	13-LnHPO	13-hydroperoxy-C18:3*n*-3	C_18_H_30_O_4_	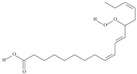	310.43 g/mol	in apple and tomato fruits [129]
5	(9*Z*,11*E*,13*E*,15*Z*)-4-oxooctadeca-9,11,13,15-tetraenoic acid	-	4-oxo-C18:4*n*-3	C_18_H_26_O_3_	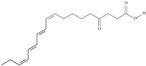	290.403 g/mol	as a methyl ester—18% of *Chrysobalanus icaco* seed oil [81]
6	(9*Z*,12*Z*,15*Z*)-2-hydroxyoctadeca-9,12,15-trienoic acid	2-Hydroxy-linolenic acid/α-hydroxylinolenic acid	2-OH-C18:3*n*-3	C_18_H_30_O_3_	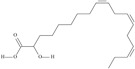	294.435 g/mol	5.4% of *Salvia nilotica* seed oil [55]
7	(9*Z*,12R,15*Z*)-12-hydroxyoctadeca-9,15-dienoic acid	densipolic acid	12-OH-C18:2*n*-3	C_18_H_32_O_3_	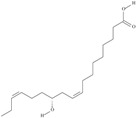	296.451 g/mol	2.1% of the seed oil of *Lesquerella auriculata* (this fatty acid is a major component of *Lesquerella* species seed oils from southeastern regions of the U.S.) [130]; the largest average content in the seeds of the eastern U.S. species: *L. perforata*, *L. stonensis*, *L. densipila*, *L. lyrata*, and *L. lescuria*—over 40% of total fatty acids [131]; in cv Linola 989 *Linum usitatissimum* (low linolenic flax) seeds at levels of 0.2 to 1% [132]
8	(11*Z*,17*Z*)-14-hydroxyicosa-11,17-dienoic acid	auricolic acid	14-OH-C20:2*n*-3	C_20_H_36_O_3_	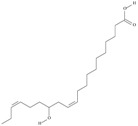	324.505 g/mol	32% of the seed oil of *Lesquerella auriculata* [130]; the largest average content in the seeds of *L. auriculata* and *L. densiflora*—over 30% of total fatty acids [131]
9	(2*E*,6*E*,10*Z*)-7-ethyl-3,ll-dimethyl-2,6,10-tridecatrienoic acid (also as a methyl ester)	-	3,11-diMe,7-Et-C13:3*n*-3	C_17_H_28_O_2_	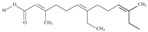	264.41 g/mol	converted to the juvenile hormon in the moth *Hyalophora cecropia* males [133]

## Abbreviations

The following abbreviations are used in this manuscript:

13-LnHPO	(9*Z*,11*E*,15*Z*)-13-Hydroperoxyoctadeca-9,11,15-trienoic acid
ALA	*alpha*-Linoleic acid
ALT	Advanced Lipid Technologies
ARA	Arachidonic acid
AUCt	Area under the (concentration-time) curve
CFA	Conjugated fatty acid
C_max_	Maximum concentration
DAG	Diacylglycerol
DHA	(4*Z*,7*Z*,10*Z*,13*Z*,16*Z*,19*Z*)-Docosa-4,7,10,13,16,19-hexaenoic acid (cervonic acid)
DPA	(7*Z*,10*Z*,13*Z*,16*Z*,19*Z*)-Docosa-7,10,13,16,19-pentaenoic acid (clupanodonic acid)
DTrE	(13*Z*,16*Z*,19*Z*)-Docosa-13,16,19-trienoic acid
EE	Ethyl esters
EFA	Essential fatty acid
EPA	(5*Z*,8*Z*,11*Z*,14*Z*,17*Z*)-Icosa-5,8,11,14,17-pentaenoic acid (timnodonic acid)
ETA	(8*Z*,11*Z*,14*Z*,17*Z*)-Icosa-8,11,14,17-tetraenoic acid
ETE	(11*Z*,14*Z*,17*Z*)-Icosa-11,14,17-trienoic acid (homo-*alpha*-linolenic acid)
FA	Fatty acid
FFA	Free fatty acid
FMLP	*N*-Formylmethionyl-leucyl-phenylalanine
HDA	(9*Z*)-12-Hydroxydodec-9-enoic acid
HDL	High-density lipoprotein
HFA	Hydroxy fatty acid
HPA	(6*Z*,9*Z*,12*Z*,15*Z*,18*Z*)-Henicosa-6,9,12,15,18-pentaenoic acid
iAUCt	Incremental area under the (concentration-time) curve
LA	Linoleic acid
LC	Long-chain
LDL	Low-density lipoprotein
MAG	Monoacylglycerol
MCFA	Medium-chain fatty acid
MUFA	Monounsaturated fatty acid
PA	Phosphatidic acid
PC	Phosphatidylcholine
PE	Phosphatidylethanolamine
PG	Phosphatidylglycerol
PKC	Protein kinase C
PL	Phospholipids
PUFA	Polyunsaturated fatty acid
rTG	Re-esterified TG
SC401	DHA and EPA ethylesters + Advanced Lipid Technologies
SCFA	Short-chain fatty acid
SDA	Stearidonic acid
SEDDS	Self-emulsifying drug delivery system
SLN	Solid lipid nanoparticles
SMEDS	Self-microemulsifying delivery system
SNEDDS	Self-nanoemulsifying drug delivery system
TG	Triacylglycerol (Triacylglyceride, Triglyceride)
VFA	Volatile fatty acid
VLCFA	Very long-chain fatty acid
WPI-GA	Whey protein isolate-gum arabic (complex)
